# Clinical and Biochemical Effects of Intra-Articular Autologous Conditioned Serum and Triamcinolone in an Equine Model of Synovitis

**DOI:** 10.3390/ani16091371

**Published:** 2026-04-29

**Authors:** Ana Velloso Alvarez, Anne Wooldridge, Fred Caldwell, Sandra Zetterström, Bruno C. Menarim, Taylor J. Towns, Emily C. Graff, Lindsey Boone

**Affiliations:** 1 Department of Animal Medicine and Surgery, Universidad CEU-Cardenal Herrera, CEU Universities, 46115 Alfara del Patriarca, Valencia, Spain; 2 Department of Clinical Sciences, Auburn University College of Veterinary Medicine, Auburn, AL 36832, USA; aaw0002@auburn.edu (A.W.); caldwfj@auburn.edu (F.C.); sandra.zetterstrom@hotmail.com (S.Z.); lhb0021@auburn.edu (L.B.); 3 Hästavdelningen, Hallands Djursjukhus, Slöinge, 31168 Falkenberg, Sweden; 4 Gluck Equine Research Center, Department of Veterinary Science, Martin-Gatton College of Agriculture, Food and Environment, University of Kentucky, Lexington, KY 40546, USA; bruno.menarim@uky.edu; 5 Department of Pathobiology, Auburn University College of Veterinary Medicine, Auburn, AL 36849, USA; townstj@auburn.edu (T.J.T.); ecg0001@auburn.edu (E.C.G.)

**Keywords:** horse, synovitis, autologous conditioned serum

## Abstract

Synovitis is a key contributor to the development of OA, and early modulation of the synovial environment may help limit downstream cartilage damage. This study compared the clinical and biochemical effects of intra-articular triamcinolone acetonide (TA) and autologous conditioned serum (ACS) in an equine model of IL-1β–induced synovitis. Six healthy adult horses were used in a crossover design involving five groups: PBS (negative control), IL-1β (positive control), IL-1β + ACS, IL-1β + TA, and an exploratory ACS-alone group administered post hoc to isolate its effects without IL-1β interference. Both TA and ACS mitigated inflammation through distinct profiles. TA was superior in reducing joint heat, swelling, and effusion. Conversely, IL-1β + ACS provided greater lameness improvement at 24, 36, and 72 h compared to IL-1β. ACS demonstrated potential chondroprotective advantages, as it did not increase synovial glycosaminoglycan (GAG) concentrations, which were highest in the IL-1β + TA group. ACS treatment resulted in significantly higher synovial total nucleated cell counts and total protein, driven primarily by monocyte enrichment. This cellular profile suggests that ACS may support the restoration of joint homeostasis. While TA remains highly effective for visual inflammatory signs, ACS offers a promising biological alternative for modulating the synovial environment and protecting cartilage during acute synovitis.

## Simple Summary

Joint inflammation is a primary driver of equine osteoarthritis; early medical intervention is essential to preserve long-term joint health. This study compared two widely used intra-articular treatments, the corticosteroid triamcinolone acetonide (TA) and the blood-derived product autologous conditioned serum (ACS) using a controlled model of temporary joint inflammation. The study included five groups: PBS (negative control), IL-1β (positive control), IL-1β + ACS, IL 1β + TA, and ACS-alone. Across the study periods, each horse received the different treatments in separate fetlock joints, with only one treatment administered per joint per period. Our results demonstrated that these therapies provide distinct benefits. TA was superior at reducing the physical signs of inflammation, including joint heat and swelling. In contrast, ACS provided higher improvement in lameness and appeared more protective of the joint environment, as it did not increase the release of markers associated with cartilage breakdown. Furthermore, ACS induced a unique cellular response characterized by an increase in helpful immune cells (monocytes), which may assist in restoring joint balance. These findings suggest that while steroids are highly effective for visible inflammation, blood-derived therapies like ACS offer a promising biological alternative that may better protect cartilage and support natural healing in the equine athlete.

## 1. Introduction

Osteoarthritis (OA) is a common and debilitating joint disease in both humans and horses, leading to significant morbidity, financial burden, and loss of athletic function. Although its pathogenesis is not fully understood, synovitis is recognized as a key driver of disease initiation and progression [1,2,3]. Cross-talk that occurs between the articular cartilage and synovial membrane during synovial inflammation is important for promoting the production of pro-inflammatory cytokines and catabolic enzymes, which destroy articular cartilage [2]. The joint innate immune system responds rapidly to inflammatory stimuli, characterized by an influx of neutrophils, macrophages, monocytes, and dendritic cells [4]. While this acute response is essential for pathogen clearance and tissue repair, persistent low-grade inflammation can overwhelm synovial homeostatic mechanisms and contribute to the development of OA [5,6,7]. However, it has been shown that this initial inflammatory response by the innate immune system is needed to promote restoration of synovial homeostasis, known as inflammatory resolution [4,8]. Macrophages, especially those within the synovial membrane, play a key role in orchestrating the inflammatory response by releasing anti-inflammatory cytokines, chemokines, and growth factors in reaction to the initial insult. These mediators mix into the synovial fluid, which is produced primarily by Type B fibroblast-like synoviocytes to maintain joint homeostasis [4,5,8].

The equine metacarpo/metatarsophalangeal (fetlock) joint is one of the most common sites for the development OA, particularly in athletic horses, where high-intensity loading leads to a high prevalence of debilitating joint disease [9,10]. To study potential therapeutics for this condition, researchers often employ models of acute reversible synovitis to better understand how rapid and effective treatment may promote innate immune cells in their response to reduce further damage to the synovial compartment. Equine-specific IL-1β is a validated tool for this purpose, as it is readily available for intra-articular administration and has been shown to reliably induce a short-term reversible inflammatory response [11].

Currently, the mainstay of intra-articular therapeutics is focused on modifying the signs of disease through the temporary reduction in inflammation via intra-articular administration of corticosteroids. However, there is concern regarding the use of these medications and their potential deleterious effects on articular cartilage metabolism [12,13]. Given our current understanding of the critical role that synovitis plays in the development of OA, treatment of synovitis should not only be directed to the modification of clinical signs (i.e., lameness) but also be directed toward modification of the synovial environment to reduce the progression of disease. Synovitis is an important inflammatory condition of the synovial environment in which appropriate therapeutic intervention could have positive long-lasting effects on the long-term health of the joint [6,14].

Autologous conditioned serum (ACS) is an intra-articularly administered biologic, derived from the patient’s blood and used by many equine practitioners to treat OA. Incubation of blood with activating surfaces (conditioning) results in increased production of important anti-inflammatory cytokines, pro-resolving mediators, and growth factors [15]. The conditioned serum contains increased concentrations of several anti-inflammatory cytokines and growth factors including interleukin-1 receptor antagonist protein (IL-1rap), interleukin-10 (IL-10), transforming growth factor-β (TGF-β), and insulin-like growth factor (IGF-1) [16]. In humans, ACS appeared to improve synovial homeostasis better than platelet-rich plasma [17]. Therefore, these anti-inflammatory cytokines and growth factors contained in equine ACS may have the potential to modulate the innate immune response of the synovium and support anabolic matrix metabolism of the articular cartilage [2,18].

Despite the emerging beneficial effects of ACS, intra-articular treatment with corticosteroids remains the mainstay of treatment for OA in human and equine medicine [19,20]. Due to the potent anti-inflammatory effect of corticosteroids, intra-articular administration is also common in the treatment of sterile synovitis. However, due to the reported deleterious time- and dose- dependent effects of corticosteroids on articular cartilage, some clinicians may turn to other articular medications such as ACS to resolve acute synovial inflammation without deleterious effects to the articular cartilage. A direct comparison of these two treatments in the face of acute sterile synovial inflammation (synovitis) has not been performed. The objectives of this study were to compare the clinical and biochemical effects of treatment with TA and ACS in a mild temporary model of IL-1β-induced synovitis within the metacarpo/metatarsophalangeal joints of six healthy horses. The hypotheses were as follows: (1) IL-1β would induce mild self-limiting synovitis in normal metacarpo/metatarsophalangeal joints (MCPJ/ MTPJ). The maximum severity of synovitis was expected at 8 post-injection hours (PIH) with resolution within 24–36 PIH, and (2) ACS and TA would improve clinical signs of synovitis and similarly protect the articular cartilage extracellular matrix, but ACS would cause a stronger anti-inflammatory effect reducing the concentration of PGE_2_ more efficiently than TA treatment.

## 2. Materials and Methods

### 2.1. Subjects

This study was performed in accordance with Institutional and NIH guidelines for the Care and Use of Laboratory Animals, and the study was approved by the Institutional Animal Care and Use Committee (IACUC) at Auburn University (protocol #2017-3043). Six adult male horses (3 Quarter horses, 2 Warmbloods, and 1 Thoroughbred, aged 14.6 ± 4.99 years) free of systemic disease from the Auburn University teaching herd were used. The horses were housed in individual stalls at the Large Animal Teaching Hospital during each 96 h experimental period. Stalls were designed to allow visual and auditory contact between horses. Between study periods, the horses were returned to pasture turnout. All horses were fed a standardized diet of grass hay and concentrates, with ad libitum access to water. Horses were not free of lameness, and a year before they had previously undergone metacarpo/metatarsophalangeal joints (MCP/MTP) intra-articular anesthesia in an earlier study, which ruled out fetlock-related lameness. To avoid additional invasive procedures, this block was not repeated, and a clinical exam with a standardized flexion test was used to confirm the absence of MCPJ/MTPJ lameness using an objective analysis of gait before and after flexion tests [21]. Baseline lameness evaluations were performed using an inertial sensor system (The Lameness Locator^®^ by Equinosis LLC, Columbia, MO, USA). Horses that exhibited a positive response to fetlock flexion based on subjective and objective assessment were excluded from the study.

### 2.2. Study Design

**Figure 1:**
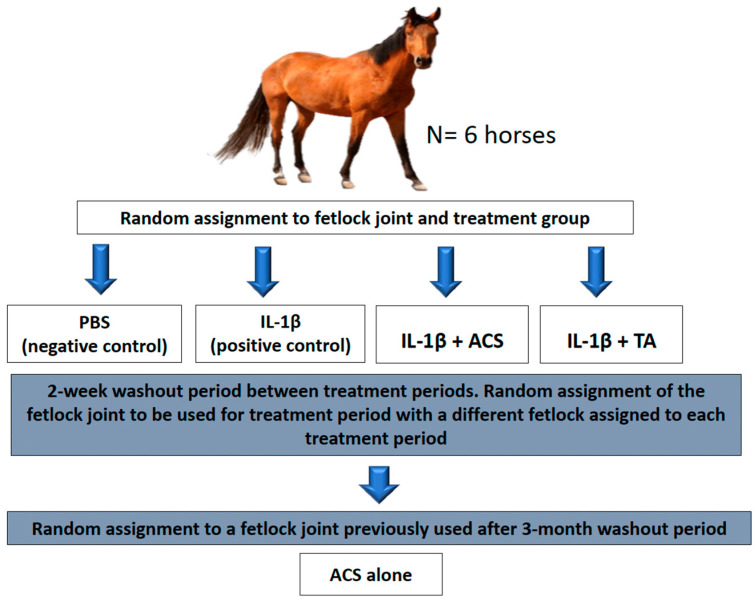
Study design. Random assignment to groups: negative control, positive control, autologous conditioned serum (ACS), and triamcinolone (TA).

The study was a blinded, 5 sequence, 5 period, and 5-treatment crossover design. Each treatment period was defined as 72 h, followed by at least 2 weeks between study periods [22]. The first 4 randomized treatments of MCP/MTP joints included: phosphate-buffered saline (PBS, negative control), IL-1β induced synovitis (positive control), IL-1β induced synovitis treated with ACS, and IL-1β induced synovitis treated with triamcinolone acetonide (TA) (Figure 1). A 14-day washout period was maintained between each of the first four crossover periods. This duration was deemed sufficient based on the transient nature of IL-1β-induced synovitis, which typically resolves within 72 h [23]. To further prevent carryover effects, a different fetlock joint was utilized for each treatment period so that no single joint received multiple inductions during the crossover. Baseline clinical and synovial parameters were confirmed to be within normal limits at the start of each period. Sequence and treatment were randomly assigned using the RAND() function in Excel for Microsoft 365 (Microsoft Corporation, Redmond, WA, USA). After analyzing the raw data regarding the synovial response to treatments by an unblinded investigator (LB), a fifth treatment group involving the same horses was created. For this group, ACS alone was administered into a randomly selected fetlock. This was elected to understand the effect of ACS without IL-1β within the joint. This treatment was administered 3 months after the last study period. Investigators (FC, JS, AW, and LB) were not blinded to treatment, but the investigators (AVA, SZ) who evaluated the clinical effects of intra-articular treatment [objective lameness evaluation and evaluating synovial response (heat, effusion, circumference)] were blinded to treatment.

### 2.3. Autologous Conditioned Serum (ACS) Preparation

ACS (Orthokine^®^; Overland Park, KS, USA) was prepared according to the manufacturer’s instructions. To minimize the potential influence of handling stress on the serum composition, ACS for each horse was produced prior to the start of the study [15]. Twenty-four hours before the first treatment period, horses were restrained in stocks, and 60 mL of blood were aseptically collected into a commercially available syringe containing glass beads. The blood was incubated at 37 °C for 24 h, centrifuged at 3000 rcf for 10 min, and the resulting serum was harvested. ACS was sterilely aliquoted into 6 mL syringes (4 mL ACS/syringe) and stored at −80 °C.

### 2.4. Intra-Articular Treatments

Synovitis was induced via intra-articular injection of recombinant equine IL-1β (R&D Systems, Inc., Minneapolis, MN, USA). To ensure a consistent inflammatory challenge throughout all study periods, all IL-1β used was from a single production lot (Lot No. OWV0316111), stored at −80 °C, and reconstituted identically according to the manufacturer’s instructions immediately prior to use.

Intra-articular injections were administered in 4–4.5 mL volumes as follows, by the unblinded investigator (LB); each group was performed individually, and a 2-week washout period was allowed between treatments.

(1) Negative control: 4 mL of sterile phosphate-buffered saline (PBS).
(2) Positive control: 100 ng equine recombinant IL-1β (R&D Systems, Minneapolis, MN, USA) diluted in 4 mL of sterile PBS to a concentration of 25 ng/mL, in a sterile fashion. This dose of IL-1β used for the study of equine synovitis caused a well-documented in vivo response [24].
(3) ACS: 4 mL of ACS injected intra-articularly.
(4) IL-1β + ACS: 100 ng of IL-1β diluted in 500 µL of PBS injected immediately prior to injection of 4 mL of ACS.
(5) IL-1β + TA: 100 ng of IL-1β diluted in 500 µL of PBS injected immediately prior to injection of 4 mg of TA (0.4 mL) mixed with 3.6 mL of sterile PBS.

### 2.5. Evaluation of Clinical Response

During the 5 study periods, the clinical response was evaluated by two investigators blinded to the treatment (AVA, SZ).

#### 2.5.1. Physical Examination

Clinical signs of pain were monitored by recording the rectal temperature, heart rate, and respiratory rate, as well as assessing non-weight-bearing lameness and abnormal limb position. These parameters were recorded every 2 h until PIH 8, every 4 h until PIH 16, and then every 12 h until PIH 72. To avoid confounding the assessment of lameness and synovial PGE_2_ concentrations, no systemic anti-inflammatory or analgesic medications were administered during the study periods. The horses were monitored intensely for signs of severe pain or distress, and rescue analgesia protocols were in place as part of the approved IACUC protocol; however, no horse required intervention.

#### 2.5.2. Metacarpo/Metatarsophalangeal (MCPJ/MTPJ) Joint Evaluation

The MCPJ or MTPJ regions were subjectively scored for heat using digital palpation at PIH 0, 8, 16, 24, 36, 48 and 72 h. Joint effusion and the maximal degree of fetlock flexion were also measured at these times. Heat was graded from 0 to 3 (0 = none, 1 = minor, 2 = moderate, and 3 = severe). Joint circumference and degrees of flexion were measured with a standard measuring tape and a protractor, respectively. In addition, a digital infrared thermometer (ThermoPro, Toronto, ON, Canada) was used to measure the temperature at three points: the synoviocentesis site and the lateral and medial regions of the dorsal pouch. As the lateral and medial sites did not differ and represent only a limited portion of the joint surface and because the synoviocentesis site consistently yielded the highest readings, this site was chosen as the primary location for subsequent analyses. Horses were kept for 10 min at room temperature inside a building to allow acclimatization before the measurements. These temperatures were compared to the skin temperature measured at the same site on the contralateral limb.

**Figure 2:**
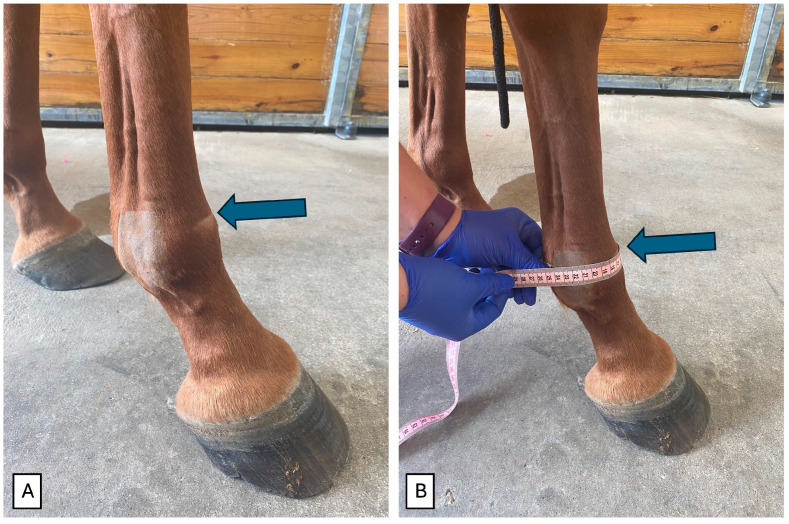
Joint circumference measurement. (**A**) Initial landmarks (blue arrow) and (**B**) measurement following the landmark.

Joint swelling was subjectively graded from 0 to 4 (0 = no swelling; 1 = minimal swelling localized to the injection site; 2 = mild swelling localized to the MCP/MTPJ; 3 = moderate swelling extending proximally toward the carpus or tarsus; and 4 = marked swelling extending to or above the carpus or tarsus). Joint circumference (mm), to evaluate joint effusion, was measured at the level of the proximal sesamoid bones, 2 cm proximal to the ergot [25], using a standard cloth measuring tape. The site for circumferential measurement was marked prior to intra-articular injection by clipping the hair away on a horizontal line on the dorsal aspect of and palmar/plantar aspect of the joint. This ensured that joint circumference was consistently measured at the same site on the limb during the study period (Figure 2).

**Figure 3:**
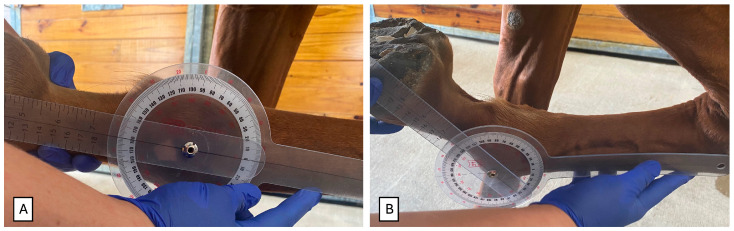
Degree of the maximum flexion was measured objectively with a protractor placed over the center of the fetlock joint. The protractor was placed over the lateral side of the joint in extension (**A**), and the joint was flexed until a pain response was noted and then measured (**B**).

Passive flexion was done until obtaining a painful reaction, and it was subjectively graded from 0 to 3 (0 = none; 1 = minor; 2 = moderate; 3 = severe). Additionally, a protractor (placed centered on the MCPJ/MTPJ) was used to objectively measure the maximum range of flexion (Figure 3). The angle measurement was obtained by flexing the fetlock until a painful response was noted and then measuring the angle at that point.

Clinical evaluations were performed on each horse prior to each study period and prior to synovial fluid collection. 

#### 2.5.3. Lameness Evaluation

Lameness was evaluated objectively using an inertial sensor system (Lameness locator, Equinosis LLC, Columbia, MO, USA) at 0, 8, 16, 24, 36, 48, and 72 h PIH. The Vector Sum data, which measure the intensity of the lameness (differences between the DiffMax and DiffMin in mm) was compared to the baseline in all the treatment groups. Because horses could show a baseline lameness unrelated to the fetlock joint, the difference in Vector Sum from baseline was calculated for each treatment.

To ensure that no residual inflammation or lameness influenced subsequent treatments, horses were clinically evaluated between each crossover period using a complete physical examination, objective lameness assessment, and standardized flexion test. No fetlock was reinjected during the initial four treatment periods. The only repeated injection occurred three months later for the ACS-alone group, at which time residual subclinical inflammation was assessed using the same clinical protocol.

### 2.6. Synovial Fluid Cytology

#### 2.6.1. Synovial Fluid Collection

At the start of each study period, horses were sedated with xylazine hydrochloride (0.02–0.8 mg/kg IV). The fetlock assigned for treatment during that study period was clipped and aseptically prepared. The collateral sesamoidean ligament approach to the metacarpophalangeal joint was used, because synovial fluid is more readily obtained uncontaminated with blood from this site [26]. Approximately 3 mL of synovial fluid were collected. Treatment syringes were prepared by unblinded investigators (LB, FC, JS) and placed in non-transparent syringes for administration. Without removing the needle, the prepared treatment was injected into the fetlock joint. Synovial fluid was collected using the same technique at PIH 8, 24, and 48 h by a blinded investigator (AVA).

#### 2.6.2. Synovial Fluid Analysis

Synoviocentesis was performed at each time point obtaining a maximum of 3 mL. The samples were divided, and 500 μL of synovial fluid was transferred to a collection tube containing EDTA for cytological analysis. The total nucleated cell count (TNCC) was measured on an automated processor (Advia 2120 hematology analyzer; Siemens Healthcare Diagnostics, Erlangen, Germany) and validated on direct cytology preparations. The total protein (TP) was measured on a handheld refractometer. Cytological findings and differential cell counts were performed by two board-certified veterinary clinical pathologists (ECG and TJT), who were blinded to the sample treatments. The remaining synovial fluid was aliquoted into Eppendorf tubes, centrifuged (600 RMP for 10 min at 4 °C), supernatant removed and stored at −80 °C until further analysis.

### 2.7. PGE_2_ Concentrations in Synovial Fluid

Thawed samples (200 μL) were hyaluronidase-digested (10 μL of 100 IU hyaluronidase/mL acetate buffer; Worthington Biochemical Corporation, Lakewood, NJ, USA) for 30 min at 37 °C, centrifuged (12,000 RPM for 10 min; 4 °C), and the supernatant was recovered. PGE_2_ was quantified by ELISA (R&D Systems, Minneapolis, MN, USA). Briefly, hyaluronidase-digested samples were solid-phase extracted (500 μL synovial fluid in 490 μL 100% ethanol and 10 μL glacial acetic acid incubated at 23 °C for 5 min), centrifuged (600 RPM for 8 min; 4 °C), and the supernatant was collected [27]. Samples were diluted at 1:20, and PGE_2_ measurements were performed in duplicate according to the manufacturer’s instructions. This colorimetric assay was not equine-specific; however, it has been previously referenced and validated for cross-reactivity in equine synovial fluid [28,29,30]. Standards provided for the ELISA were used to prepare a standard curve following the manufacturer’s instructions.

### 2.8. 1,9-Dimethyl Methylene Blue Assay (DMMB)

The DMMB assay was performed to measure the GAG concentration in the synovial fluid. Synovial samples were digested with hyaluronidase. One aliquot of each sample was digested by incubation with 10 units of *Streptomyces* hyaluronidase (*Streptomyces* hyaluronidase, Calbiochem, San Diego, CA, USA) at 34 °C for 1 h, as previously described, and the supernatant was recovered [31]. The 1,9-dimethyl methylene blue dye (Sigma-Aldrich, St. Louis, MO, USA) was prepared following the method of Farndale et al. [32]. The standard curve was created by preparing solutions containing 0 to 65 µg of chondroitin sulfate (Lot SLCB0351, Sigma-Aldrich, St. Louis, MO, USA). Briefly, using a 96-well flat-bottom transparent plate (Stellar Scientific, Baltimore, MD, USA), 50 µL of diluted digested synovial fluid (1:10 dilution in distilled water) were mixed with 200 µL of DMMB dye (1 mN hydrochloric acid, 0.06‰ DMMB, 40 mM glycine, 27 mM NaCl pH 3), and the plate was shaken on a horizontal orbital microplate shaker (0.12″ orbit) at 500 RPM for 5 s. Measurement of the total GAG content was performed by a direct spectrophotometric method. Optical density was measured at 525 nm on a microplate reader (SpectraMax ID3, Molecular Devices, Sunnyvale, CA, USA) [31]. Samples were measured in triplicate.

### 2.9. Statistical Analysis

Prior to the start of the study, a power analysis was performed (G*Power v. 3.1) to determine the required sample size. Based on previously published data using the 100 ng recombinant equine IL-1β induction model, an effect size of 1.68 was estimated for the primary outcome measures (PGE_2_ concentration and objective lameness) [22,23,24]. This large effect size reflects the robust and consistent inflammatory response characteristic of this model relative to the low variability observed in standardized experimental equine populations. With an alpha of 0.05 and a power (1 − β) of 0.80, it was determined that a sample size of n = 6 horses was sufficient to detect statistically significant differences between treatment groups.

Linear or generalized linear mixed models were used to analyze each clinical or biochemical variable. Timepoints with some or all treatments with all 0 responses were not included in the analyses, as this caused the model not to converge (e.g., timepoint 0 for heat and swelling, timepoint 0 and 72 for joint effusion and timepoint 0, 36 and 72 for pain to flexion). White blood cell counts, %lymphocytes and red blood cell counts + 1 were natural log transformed prior to prevent violation of homogeneity of variance.

The generalized linear mixed models (GLMM) for heat, swelling, joint effusion and pain to flexion included fixed factors for treatment, time, resident and a treatment by time interaction effect. A multinomial distribution was assumed with a cumulative logit (ordered) link function. While the ACS-alone group was administered after the initial four crossover periods, the inclusion of random effects for ‘horse’ and ‘fetlock’ statistically controlled for individual animal variability. Due to its post hoc addition, all comparisons involving the ACS-alone group are considered exploratory.

The linear mixed models (LMM) for angle, circumference, heart rate, temperature, respiratory rate, white blood cell count, total protein, red blood cell count, specific gravity, %neutrophils, %monocytes, %lymphocytes, % eosinophils, PGE2, GAG, front limb lameness, hind limb push lameness and hind limb impact lameness included fixed factors for treatment, time and a treatment by time interaction effect. The LMM for temperature on limb included fixed factors for treated (yes/none), location, time and treatment and all 2-, 3- and one 4-way interaction effects.

Random intercepts were included in all GLMMs, and LMMs for each horse and fetlock within each horse were included to account for within-horse and within-fetlock correlations. Model residuals for LMMs were examined to evaluate the assumption of normality. Simple effects were tested to compare treatments at each time, and multiple comparisons were adjusted for using Tukey’s test. The Satterthwaite degrees of freedom method was used in all models.

To evaluate the clinical magnitude of treatment effects beyond statistical significance, standardized effect sizes were calculated for primary clinical and biochemical variables using Hedges’ *g*. This metric was selected to account for the small sample size (n = 6), with effect sizes interpreted as small (0.2), medium (0.5), and large (≤0.8) (Table S1).

## 3. Results

### 3.1. Clinical Parameters

**Figure 4:**
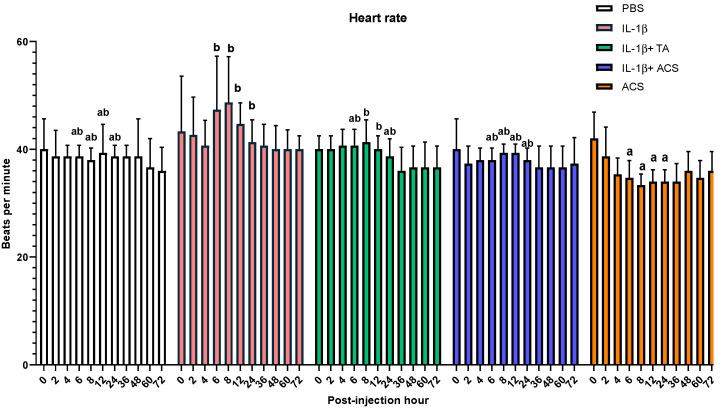
Heart rate after injecting a fetlock joint with phosphate-buffered saline (PBS), equine recombinant IL-1β (IL-1β), IL-1β + triamcinolone (TA), IL-1β + autologous conditioned serum (ACS), or ACS. Different letters indicate a significant difference (*p* < 0.05) between groups at 6, 8, 12 and 24 PIH. Bar graphs indicate the mean and standard deviation.

No differences between treatments for the respiratory rate (*p* = 0.431) or rectal temperature (*p* = 0.717) were observed for all time points. Differences in the heart rate between ACS alone and PBS, IL-1β, IL-1β + TA, and IL-1β + ACS groups were observed at 6, 8, 12 and 24 h PIH (Figure 4). At 6, 8, 12 and 24 PIH, the heart rate was lower in the group receiving ACS compared to IL-1β alone (*p* = 0.03, *p* = 0.001, *p* = 0.02 and *p* = 0.01, respectively). At 8 and 12 PIH, the ACS group had a lower heart rate compared to the IL-1β + TA group (*p* = 0.001 and *p* = 0.03).

### 3.2. Metacarpo/Metatarsophalangeal Joint (MCPJ/MTPJ) Evaluation

**Figure 5:**
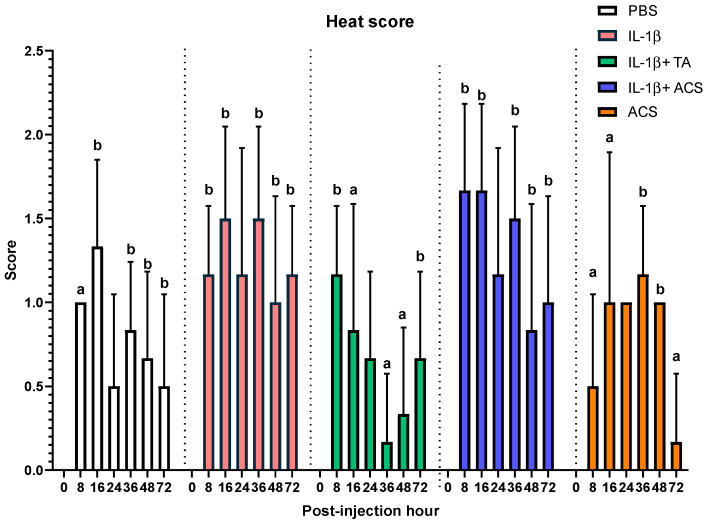
Cutaneous heat scores after injecting a fetlock joint with phosphate-buffered saline (PBS), equine recombinant IL-1β (IL-1β), IL-1β + triamcinolone (TA), IL-1β + autologous conditioned serum (ACS), or ACS. Heat was graded from 0 to 3 (0 = none, 1 = minor, 2 = moderate, and 3 = severe). Different letters indicate a significant difference (*p* < 0.05) between groups at 8, 16, 36, 48 and 72 PIH. Bar graphs indicate the mean and standard deviation.

The skin over the joints injected with IL-1β, IL-1β + TA, and IL-1β + ACS had a higher temperature than the skin of the contralateral joint at all the time points (*p* < 0.05). Differences in heat scores between treatment groups were observed at 8, 16, 36, 48, and 72 h post-injection (Figure 5). Eight hours post-injection, the cutaneous heat score in the ACS group was lower than the cutaneous heat scores of the PBS, IL-1β, and IL-1β + ACS groups (*p* = 0.001). At 16 h, the IL-1β + TA group had a lower cutaneous heat score compared to the PBS (*p* = 0.04), IL-1β (*p* = 0.01) and IL-1β + ACS (*p* = 0.03) groups. At 36 and 48 h post-injection, the IL-1β + TA group still had a lower cutaneous heat score than all treatment groups (*p* = 0.001), though at 72 h, the ACS group had a significantly lower cutaneous heat score than all the other groups (*p* = 0.001).

**Figure 6:**
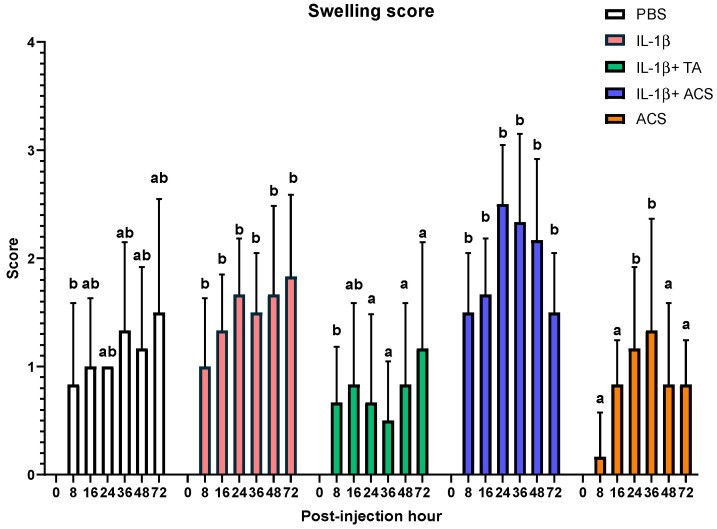
Swelling score after injecting a fetlock joint with phosphate-buffered saline (PBS), equine recombinant IL-1β (IL-1β), IL-1β + triamcinolone (TA), IL-1β + autologous conditioned serum (ACS), or ACS. Graded from 0 to 4 (0 = no swelling; 1 = minimal swelling localized to the injection site; 2 = mild swelling localized to the MCP/MTPJ; 3 = moderate swelling extending proximally toward the carpus or tarsus; and 4 = marked swelling extending to or above the carpus or tarsus). Different letters indicate a significant difference (*p* < 0.05) between groups at 8, 16, 24, 36, 48 and 72 PIH. Bar graphs indicate the mean and standard deviation.

Swelling was more severe at 72 h in the IL-1β, and IL-1β + ACS compared to the IL-1β + TA and ACS groups, while the PBS group did not differ from any treatment. Differences in the swelling scores between treatment groups were observed at 8, 16, 24, 36, 48, 72 h post-injection (Figure 6). Eight hours post-injection, the swelling score in the ACS group was lowest compared to the rest of the groups (*p* = 0.001). At 16 h, the ACS group had decreased swelling compared to IL-1β (*p* = 0.03) and IL-1β + ACS (*p* = 0.04). At 24 and 36 h post-injection, IL-1β + TA had lower swelling scores compared to IL-1β (*p* = 0.001 and *p* = 0.003), IL-1β + ACS (*p* = 0.001 for both time points), and ACS (*p* = 0.01 and *p* = 0.03) groups. The groups IL-1β + TA and ACS had lower swelling scores compared to the IL-1β and IL-1β + ACS groups at 48 h (*p* = 0.001) and 72 h (*p* = 0.002).

**Figure 7:**
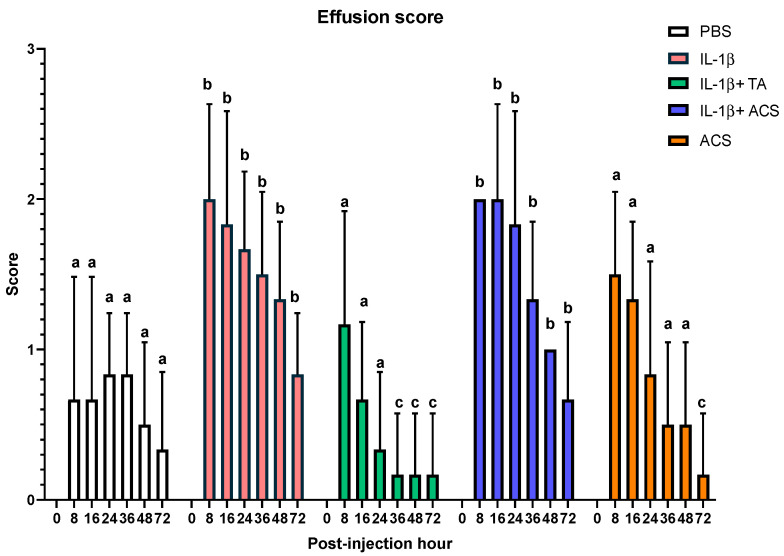
Joint circumference scores after injecting a fetlock joint with phosphate-buffered saline (PBS), equine recombinant IL-1β (IL-1β), IL-1β + triamcinolone (TA), IL-1β + autologous conditioned serum (ACS), or ACS. Different letters indicate a significant difference (*p* < 0.05) between groups at 8, 16, 24, 36, 48 and 72 PIH. Bar graphs indicate the mean and standard deviation.

Differences in joint effusion scores between treatment groups were observed at 8, 16, 24, 36, 48, 72 h post-injection (Figure 7). At all time points post-injection, the IL-1β and IL-1β + ACS had more joint effusion than other groups (*p* = 0.001). IL-1β + TA group had the lowest joint effusion scores at 36 and 48 PIH (*p* = 0.001).

No differences in pain response to flexion were found between groups at any time point. When measuring the range of flexion with a protractor, the IL-1β group had a reduced range of flexion compared to IL-1β + TA (*p* = 0.04) and ACS (*p* = 0.03) at 48 h.

### 3.3. Lameness Evaluation

**Figure 8:**
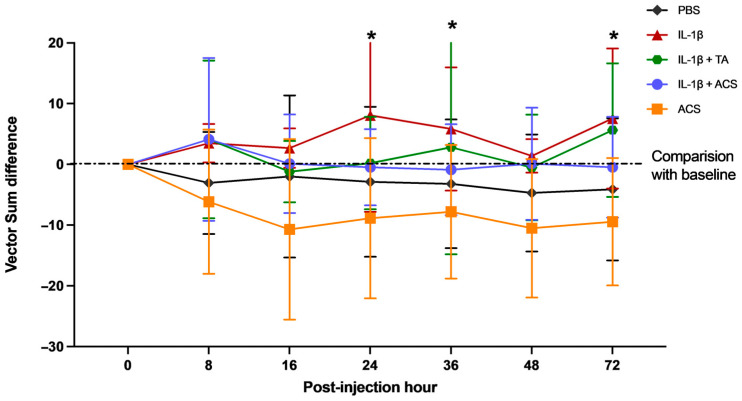
Lameness measured objectively compared to baseline after injection of phosphate-buffered saline (PBS), equine recombinant IL-1β (IL-1β), IL-1β + triamcinolone (TA), IL-1β + autologous conditioned serum (ACS), or ACS treatment groups. The symbols represent the mean and the error bars the standard deviation. * denotes a significant difference between groups at the same time point, *p* < 0.05.

The lameness score (vector sum value) obtained for each time point was compared to the baseline, and each treatment group was compared at 8, 16, 24, 36, 48 PIH (Figure 8). In the groups where IL-1β was injected alone or combined with TA or ACS, a mild to moderate increase in lameness was observed (12.5 ± 3.22 mm). Injection of IL-1β + ACS induced less lameness than injection of IL-1β alone at 24, 36, and 72 PIH (*p* = 0.005; Hedges’ *g* = 1.32, 0.005, and 0.01 respectively). Curiously, for all horses whose joints were injected with ACS alone, the lameness improved from the baseline compared to each time point. When comparing the IL-1β + TA and IL-1β + ACS groups, lameness was less marked with the addition of ACS to IL-1β compared to the addition of TA to IL-1β at 36 and 72 PIH (*p* = 0.02 and 0.03).

### 3.4. Synovial Fluid Cytology

**Figure 9:**
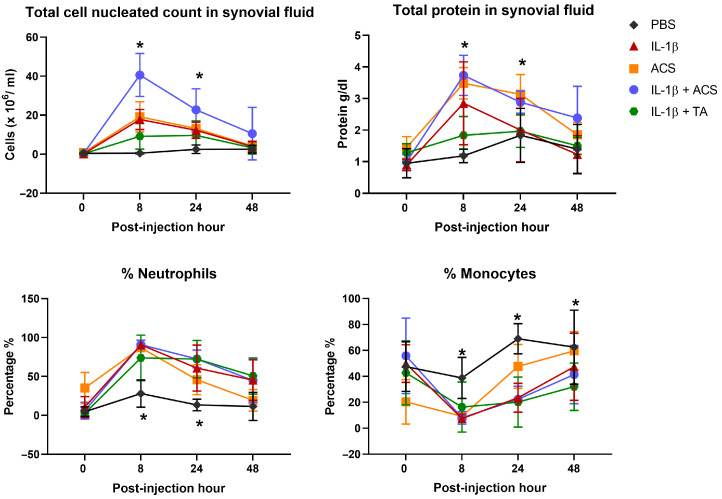
Synovial fluid analysis parameters after injection phosphate-buffered saline (PBS), equine recombinant IL-1β (IL-1β), IL-1β + triamcinolone (TA), IL-1β + autologous conditioned serum (ACS), or ACS treatment groups. The symbols represent the mean and the error bars the standard deviation. * denotes a significant difference between groups at the same time point, *p* < 0.05.

Synovial fluid was obtained from all horses for all study periods and time points (Figure 9). At 8 and 24 PIH, the TNCC was higher in all groups compared to PBS (*p* = 0.01). At 8 PIH, horses injected with IL-1β, IL-1β + ACS, and ACS alone had a significant increase in TNCC compared to PBS and IL-1β + TA (*p* < 0.001) that gradually decreased over time, with no significant difference noted at 48 PIH. This increase was primarily owing to an increase in neutrophils, which were significantly lower in the PBS treatment group compared to the remaining treatments (*p* < 0.001). At 24 and 48 PIH, the percentage of monocytes was increased in the ACS treatment group compared to the IL-1β, IL-1β + TA, and IL-1β + ACS groups (*p* = 0.001). When comparing between groups, the IL-1β + ACS group produced the highest TNCC (40,625 ± 11.01 cells/µL; *p* = 0.001, Hedges’ *g* = 2.14). Similarly, IL-1β, IL-1β + ACS, and ACS alone had a significant increase in TP in synovial fluid at 8 PIH compared to the baseline (*p* < 0.001). This increase in TP remained in the IL-1β + ACS and ACS groups at 24 PIH (*p* = 0.001; Hedges’ *g* = 1.33 and 0.01 respectively), but no differences between groups were observed at 48 PIH.

### 3.5. PGE_2_ Concentrations in Synovial Fluid

**Figure 10:**
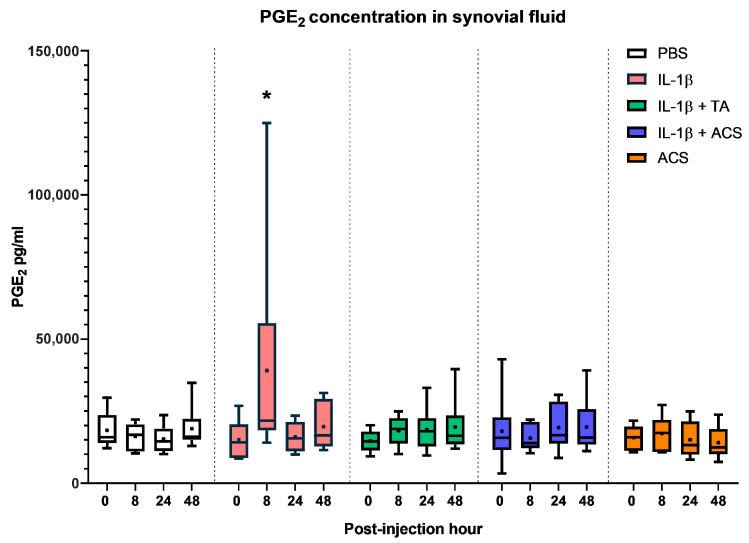
PGE2 concentrations in the synovial fluid at 0, 8, 24, and 48 h after injecting a fetlock joint with phosphate-buffered saline (PBS), equine recombinant IL-1β (IL-1β), IL-1β + triamcinolone (TA), IL-1β + autologous conditioned serum (ACS), or ACS. The boxplots represent the interquartile range (IQR) of n = 6. The black lines represent the median values, the black dots represent the mean, and the whiskers represent the values outside the IQR. * denotes a significant difference between IL-1β group at 8 PIH compared to the rest of the groups at the same time point, *p* < 0.05.

PGE_2_ synovial fluid concentration was evaluated at 0, 8, 24, and 48 PIH (Figure 10) for all the groups. PGE_2_ was increased by 1.6-fold in the IL-1β group (*p* < 0.001) compared to the baseline, but no significant increase in PGE_2_ concentration was observed for all other treatments at any time point. One horse, after injection of IL-1β, had an increase of PGE2 5.7 times higher at 8 PIH than the average PGE2 of five other horses in the same group.

### 3.6. 1,9-Dimethyl Methylene Blue Assay (DMMB)

**Figure 11:**
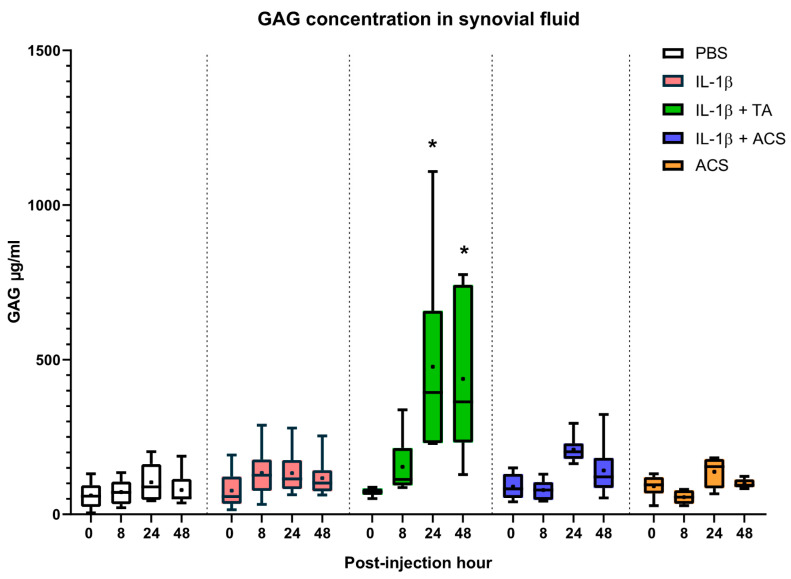
GAG concentrations in the synovial fluid at 0, 8, 24, and 48 h after injecting a fetlock joint with phosphate-buffered saline (PBS), equine recombinant IL-1β (IL-1β), IL-1β + triamcinolone (TA), IL-1β + autologous conditioned serum (ACS), or ACS. The boxplots represent the interquartile range (IQR) of n = 6. The black lines represent the median values, black dots represent the mean, and the whiskers represent the values outside the IQR. * denotes a significant difference between IL-1β + TA group at 24 and 48 PIH compared to the rest of the groups at the same time point, *p* < 0.05.

GAG concentration was measured in synovial fluid at 0, 8, 24, and 48 PIH (Figure 11). No significant difference was observed in the median GAG concentration in the PBS, IL-1β, IL-1β + ACS, and ACS groups during any time points. When comparing treatment groups, an increased concentration of GAG was measured in the IL-1β + TA group at 24 and 48 PIH (*p* < 0.001; Hedges’ *g* = 1.45).

## 4. Discussion

The primary pre-specified hypothesis of this study, that intra-articular ACS would reduce synovial PGE_2_ concentrations more efficiently than TA, was not confirmed. Both treatments were equally effective at mitigating the IL-1β-induced PGE_2_ rise; by 8 PIH, concentrations in both groups returned to levels indistinguishable from the negative (PBS) control. Despite these similar effects on PGE_2_, the two treatments produced distinct effects on clinical parameters, synovial fluid cellularity, and GAG concentrations. IL-1β + ACS generated the most pronounced neutrophilic response in synovial fluid, yet this treatment produced the most significant improvement in lameness. In contrast, while IL-1β + TA was more effective at reducing heat, swelling, and effusion, it resulted in a higher GAG concentration at 24 and 48 h. This increase in soluble GAG likely reflects elevated matrix turnover or acute catabolic activity associated with TA, whereas the stability of GAGs in the ACS-treated groups suggests a more favorable immediate metabolic profile for the articular cartilage.

These findings highlight a dissociation between the visual suppression of inflammation and the functional restoration of the joint. Additionally, the significantly lower heart rate observed in the ACS-alone group compared to the IL-1β challenged groups likely reflects a reduction in sympathetic activation. Since the horses in the ACS-alone group did not receive the pro-inflammatory IL-1β stimulus, they did not experience the systemic stress response or tachycardia typically associated with acute synovitis pain.

Synovitis initiates a cytokine cascade capable of driving cartilage matrix destruction and OA progression [3,33]. While TA and ACS have been evaluated in healthy and OA-affected equine joints [28,34,35,36], their effects have not previously been compared in an in vivo synovitis model. The primary goal of intra-articular therapies is to modulate inflammation and protect cartilage. ACS has demonstrated disease-modifying properties in both human and equine studies by reducing clinical signs of joint disease and reducing the cellular response in synovial fluid, increasing the concentration of IL-1rap in synovial fluid, and improving histologic scores in the synovial membrane [28,37,38,39].

In this study, the IL-1β + ACS group exhibited a robust cellular influx, reaching a TNCC of 40,625 ± 11.01 cells/μL. In a non-experimental clinical setting, a TNCC of this magnitude (where normal fetlock ranges are ~200–500 cells/μL) would traditionally raise concerns for septic arthritis [40]. However, the differential was predominantly monocytic rather than degenerate neutrophilic. Crucially, this response did not cause lameness; instead, the lameness scores improved relative to the baseline. Our findings align with an emerging paradigm: that transient cellular influx following orthobiologic administration represents a “pro-resolving” homeostatic response rather than harmful inflammation [41,42,43], given the observed macrophage profile, this likely reflects an active homeostatic process [8].

A key exploratory finding was the marked increase in synovial GAG concentrations in the IL-1β + TA group, which was not observed in the IL-1β-only or ACS-treated groups. This aligns with previous evidence that corticosteroids, while potent anti-inflammatories, can exert immediate catabolic effects on the cartilage extracellular matrix, potentially accelerating the release of proteoglycans into the synovial fluid [44,45] While TA effectively suppressed effusion and heat, the biochemical data suggest it may simultaneously increase matrix turnover or induce acute catabolic leakage in contrast to the stability of GAGs in the ACS-treated groups. However, this interpretation must remain cautious; the increase likely reflects acute metabolic shifts rather than definitive evidence of irreversible damage. Given our 72 h window and sample size, it is premature to conclude that ACS offers better long-term “chondroprotection,” but these findings warrant longitudinal investigation in chronic OA models [20,46]. Meta-analysis indicates that while low-dose TA may be chondroprotective, higher cumulative doses (over 18 mg) can be detrimental, downregulating collagen and aggrecan gene expression [34,47]. The superior functional outcome of ACS aligns with human clinical trials where ACS provided superior pain relief compared to TA for hip OA or lumbar radicular compression [48,49]. While no previous head-to-head comparisons existed in equine induced-synovitis models, our results mirror those of Jostingmeir et al., who reported a high lameness improvement in horses with distal interphalangeal OA treated with ACS compared to corticosteroids combined with hyaluronic acid [50].

IL-1β was selected for this model because it is a key pro-inflammatory cytokine involved in OA pathogenesis across multiple equine joints [11,22,23,24,51,52], where PGE_2_ and GAG concentrations are commonly measured as indicators of inflammation [23,24,53,54]. While increased concentrations of PGE_2_ and GAG have been associated with repeated arthrocentesis in earlier work [55], this phenomenon was not observed in the present study. The PBS group showed no significant deviations in cytology, PGE_2_, or GAG across 72 h, confirming that our results were true reflections of the treatments rather than artifacts of sampling.

Previous studies using an IL-1β synovitis model in the middle carpal joint showed higher synovial cell concentrations at 8 h (170.70 ± 37.58 cells/uL) compared with the fetlock response observed here (17.80 ± 5.12 cells/uL) [24]. This 10-fold lower TNCC suggests that the metacarpo/metatarsophalangeal (MCP/MTP) joint may be more resistant to IL-1β induction than the carpus. This “floor effect” likely attenuated the therapeutic signal, potentially making it more difficult to detect statistical superiority between the two treatments. Our findings align with research by Colbath et al., which demonstrated that an identical intra-articular dose of IL-1β elicits significantly different responses depending on the joint involved [23]. Furthermore, substantial inter-horse variability is frequently observed in equine research across outcome measures, including lameness severity and cytokine concentrations [11,56]. While the heterogeneity of our population (diverse breeds; mean age 14.6 years) may have contributed to this variability, the randomized crossover design effectively allowed each horse to serve as its own control. Additionally, the variability in response to IL-1β between individuals and manufacturing concerns such as different lots, methods of reconstitution, and storage has been reported to lead to varying levels of activity of intra-articularly administered IL-1β [22,23,24,52]. For the present study, all IL-1β administered was from the same lot (Lot. Number OWV0316111) and stored and reconstituted identically. Ultimately, the successful induction of mild synovitis was confirmed by the documented increases in clinical parameters (lameness, effusion, heat) and synovial fluid biomarkers (PGE_2_ and GAG).

PGE_2_ is a well-recognized pro-inflammatory mediator within the joint [57]. Elevated PGE_2_ and GAG have been correlated with OA-associated changes in experimentally induced OA in horses [28]. In this study, the PGE_2_ concentrations did not rise following IL-1β combined with ACS or TA, suggesting both treatments mitigated IL-1β–induced inflammatory signaling. This aligns with prior in vitro data demonstrating that ACS significantly decreased PGE_2_ production in IL-1β-stimulated explants, whereas TA did not [58]. Consistent with these findings, intra-articular ACS reduced both lameness scores and PGE_2_ concentrations compared to PBS in a previous in vivo study [28]. In the present study, reduced lameness was correlated with decreased PGE_2_ concentration. Recent work evaluating APS in an IL-1β-induced synovitis model further supports this relationship: although APS did not significantly improve lameness or synovial fluid parameters, it did reduce the gross and histopathology scores, indicating a beneficial effect on joint tissues despite minimal changes in clinical signs [56]. Together, these findings suggest that biological therapies such as ACS may exert protective or disease-modifying effects within the inflamed synovial environment, even when short-term clinical improvements are modest.

An additional challenge in orthobiologic studies is the potential variability in ACS efficacy, stemming from the inherent differences in cytokine composition that have been documented in horses [16], and surgical stress has been shown to alter ACS cytokine concentrations [15]. To minimize this, ACS was prepared for each horse before the study began. The initial experimental design did not include the use of ACS alone, but because of a marked increase in the TNCC and TP observed in the IL-1β + ACS group, unblinded investigators chose to evaluate the intra-articular effect of ACS when administered alone. A significant limitation in evaluating these disease-modifying effects was the lack of IL-10 measurement within the synovial fluid. Inflammatory resolution is no longer viewed as a passive event but as an active process orchestrated primarily by macrophages to restore joint homeostasis [59,60]. Characterizing the anti-inflammatory cytokine profile would have clarified whether the robust predominantly monocytic response observed after ACS administration was definitively pro-resolving [5,14]. Evaluating the correlation between this monocyte increment and homeostatic markers remains a priority endpoint for follow-up studies to confirm that ACS-induced cellularity represents a beneficial protective mechanism rather than detrimental inflammation.

Several aspects of the study design warrant careful interpretation. First, ACS and TA were administered concurrently with IL-1β, meaning that synovitis was not allowed to peak before treatment. Consequently, this model does not fully replicate naturally occurring clinical synovitis, in which inflammation is already established prior to intervention. Instead, the present design primarily evaluates the ability of ACS and TA to modulate or mitigate the early inflammatory cascade triggered by IL-1β. Second a 14-day washout period was chosen based on the known transient nature of IL-1β-induced synovitis, where parameters typically normalize within 72 h. While we cannot entirely rule out subclinical cytokine priming or altered cell trafficking, no significant differences were observed in the baseline clinical or biochemical parameters between study periods. Furthermore, the randomized crossover design effectively mitigated the potential impact of any carryover effects on the final analysis.

Finally, it is important to highlight that the ACS-alone group was added as an outcome-adaptive modification to the original study design. Because this group did not undergo the full randomized crossover sequence, these results should be viewed as exploratory. In this group, a mild improvement in lameness was observed compared with the baseline. While all horses underwent standardized exams and flexion tests at enrollment to exclude clinically relevant fetlock-associated lameness, very mild or subclinical changes cannot be entirely ruled out, given the time elapsed since the original diagnostic work-up. Nevertheless, the consistent improvement is more likely attributable to the known analgesic and anti-inflammatory effects of ACS, though minor age-related joint changes may have contributed. To build upon these findings, future high-powered pre-registered studies should evaluate different IL-1β dosages for inducing synovitis in the fetlock. Incorporating a broader cytokine panel and additional longitudinal biomarkers, such as CPII and CS 846, will be crucial to further elucidate the complex intra-articular effects and homeostatic potential of TA and ACS.

## 5. Conclusions

Intra-articular administration of ACS alone produced a mild and transient inflammatory response, characterized by increased joint heat, swelling, effusion, and elevated TNCC. Importantly, this response did not cause lameness; instead, the lameness scores improved relative to baseline. In horses with synovitis, ACS appeared to provide more clinical benefit than TA. Although both treatments reduced PGE_2_ production, ACS did not increase GAG concentrations in synovial fluid, suggesting a reduced impact on cartilage catabolism compared with the potentially deleterious effects associated with intra-articular corticosteroids. These findings support the potential chondroprotective advantages of ACS in the management of acute synovitis. Further research is needed to clarify how biological therapeutics modulate the acutely inflamed synovial environment and to optimize their clinical application.

## Data Availability

All data included in the manuscript are available upon editors’ request.

## Supplementary Materials

The following supporting information can be downloaded at https://www.mdpi.com/article/10.3390/ani16091371/s1, Table S1: Standardized effect sizes (Hedges’ *g*) for primary clinical and synovial fluid variables at the peak of inflammation (24 h).
