# Supplementary material for: Clinical and Biochemical Effects of Intra-Articular Autologous Conditioned Serum and Triamcinolone in an Equine Model of Synovitis
Source: Animals (Basel). 2026 Apr 29;16(9):1371. doi: 10.3390/ani16091371 (PMC13163084; doi:10.3390/ani16091371)
Supplement: Supplementary file 1 [file animals-16-01371-s001.zip › animals-4229204-supplementary.pdf]

Table S1. Standardized effect sizes (Hedges' *g*) for primary clinical and synovial fluid variables at the peak of inflammation (24 h)

Outcome Variable	ACS+IL-1β vs. IL-1β	TA+IL-1β vs. IL-1β
Lameness	1.32	1.91
Synovial TNCC	2.14	2.34
Synovial PGE2	1.41	1.62
Synovial GAG	1.36	1.45
Total Protein	1.33	1.48

Hedges' *g* was calculated to provide a standardized measure of treatment effect magnitude. Interpretation is based on established benchmarks where $g \geq 0.8$ is considered a large effect. Comparisons are relative to the IL-1β group (positive control).
